# Translation regulatory long non-coding RNA 1 (TRERNA1) sponges microRNA-23a to suppress granulosa cell apoptosis in premature ovarian failure

**DOI:** 10.1080/21655979.2021.2023802

**Published:** 2022-01-16

**Authors:** Lili Zhang, Bin Mao, Xiaodong Zhao, Yue Yuan, Wei Wang, Shaohua Lin

**Affiliations:** a Key Laboratory of Reproductive Medicine and Embryo, The Reproductive Medicine Center of the First Hospital of Lanzhou University, Lanzhou City, Gansu Province, China; b Reproductive Department of Guangxi International Zhuang Medical Hospital, Nanning City, Guangxi Province, China

**Keywords:** TRERNA1, miR-23a, premature ovarian failure, apoptosis

## Abstract

Translation regulatory long non-coding RNA 1 (TRERNA1) plays critical roles in cancer biology. We predicted the direct interaction of TRERNA1 with microRNA (miR)-23a, which promotes granulosa apoptosis. Granulosa apoptosis is involved in premature ovarian failure (POF). This study was therefore carried out to explore the involvement of TRERNA1 and miR-23a in POF. The expression of TRERNA1 and miR-23a in POF and control groups were detected by RT-qPCRs. The subcellular locations of TRERNA1 in granulosa cell line COV434 was detected by subcellular fractionation assay. The interaction between TRERNA1 and miR-23a was predicted using IntaRNA2.0. The direct interaction between COV434 and miR-23a was explored with RNA pull-down assay. In granulosa cells, the direct interaction between TRERNA1 and miR-23a was verified by overexpression assay. Cell apoptosis assay was performed to evaluate cell apoptosis. Both TRERNA1 and miR-23a were downregulated in POF. In addition, TRERNA1 was detected in both cytoplasm and nuclear samples of granulosa cells, and directly interacted with miR-23a. TRERNA1 did not affect the expression of miR-23a in granulosa cells, while TRERNA1 suppressed the role of miR-23a in enhancing cell apoptosis. In conclusion, TRERNA1 may sponge miR-23a to suppress granulosa cell apoptosis in POF.

## Background

As a common clinical disorder mainly affects females younger than 40 years old, premature ovarian failure (POF) often develops when the production of eggs ovaries is stopped [1]. Although POF can be easily diagnosed and misdiagnosis is rare, POF in many cases causes serious health consequences, such as osteoporosis, infertility, ischemic heart, autoimmune disorders and psychological distress [2,3]. It is estimated that POF affects about 1 out of 1,000 women younger than 30 years old, and 1 out of 100 women between 30 and 40 years old [4,5]. At present, there are no proven treatments for POF [6,7]. And anti-POF therapies, such as hormone replacement therapy, can only be applied to relieve symptoms and reduce health risks [6,7]. Therefore, the development of novel therapies for POF is of great significance.

The unclear molecular mechanism of POF is the main challenge for the development of novel therapies [8,9]. Certain molecular factors with critical functions in POF, such as MT1 and TGF-β-like factors, have been demonstrated to be potential molecular targets to develop targeted therapy for POF [9,10]. However, targeted therapy for many human diseases, including POF, is still under research. Previous studies have revealed that, the expression of non-coding RNAs (ncRNAs), such as long ncRNAs (lncRNAs) and microRNAs (miRNAs), are frequently altered in POF, and the regulation of certain ncRNAs might be applied to treat POF [11,12]. Zhao *et al*. reported that overexpression of lncRNA HOTAIR promoted premature ovarian failure through upregulating Notch-1 [13]. With the increased elucidation of the molecular mechanisms of POF, more molecular players with critical functions have been identified [14,15]. It has been reported that translation regulatory lncRNA 1 (TRERNA1) plays critical roles in cancer biology [16,17]. TRERNA1 promotes epithelial-mesenchymal transition and regulates gene methylation to promote cancer progression [16,17]. However, the participation of TRERNA1 in other diseases, such as POF, is unknown. We predicted that miR-23a may directly bind to TRERNA1. MiR-23a is known to promote granulosa apoptosis by targeting SMAD5 [18]. It is known that granulosa apoptosis is involved in POF [19]. We therefore speculated that TRERNA1 may interact with miR-23a to participate in POF. This study was therefore caried out to explore the involvement of TRERNA1 and miR-23a in POF. We found that TRERNA1 was downregulated in POF and inhibited cell apoptosis, possibly by upregulating miRNA-487a.

## Materials and methods

### Research patients

A total of 50 POF patients were recruited at the Guangxi International Zhuang Medical Hospital. The Ethics Committee of this hospital approved this study. All experimental procedures were carried out in accordance with the World Medical Association Declaration of Helsinki. All controls received infertility treatment due to tubal obstruction or male factors. POF patients were treated with embryo transfer. Granulosa cell (GC) tissues were collected during treatment. The accepted criteria of POF: the occurrence of sporadic menstruation or amenorrhea for more than 4 months before the age of 40, and the FSH level was greater than 40 IU/L twice. The baseline data of participants were presented in Table 1.

**Table 1.** Participants’ clinical data

Variables	Control (n = 50)	POF (n = 50)
Age (years)	29.66 ± 3.99	29.34 ± 3.89
Basal FSH (IU/L)	6.89 (5.89, 8.13)	15.48 (12.07, 22.37)**
BMI (kg/m2)	23.11 (18.72, 24.34)	22.45 (19.01, 25.99)
Basal LH (IU/L)	5.62 (3.67, 7.99)	5.63 (4.12, 8.99)
Basal E2 (pg/mL)	28.99 (23.23, 41.69)	30.12 (12.11, 42.98)
AMH (ng/mL)	3.21 (2.11, 5.45)	0.48 (0.24, 0.91)**

**p < 0.01.

### Cell culture and transfections

Human granulosa-like KGN cells (tumor cells, RIKEN BioResource Center) were used to perform *in vitro* experiments. Cell culture was performed in DMEM/F-12 medium (Gibco, USA) containing both 10% fetal bovine serum (FBS, Sigma-Aldrich; Merck KGaA, Darmstadt, Germany) and 1% penicillin/streptomycin. A 95% humidity incubator was used to perform cell culture with the temperature and CO_2_ set to 37°C and 5%, respectively.

The overexpression of TRERNA1 (NCBI Accession: NR_051976.1) and miR-23a (5ʹ-AUCACAUUGCCAGGGAUUUCC-3ʹ) was achieved in cells using Lipofectamine 2000 (Thermo Fisher Scientific, Beijing, China). Overexpression was performed by transfecting TRERNA1 vector (pcDNA 3.1, Invitrogen) and/or miR-23a mimic (Sigma-Aldrich) into 10^7^ cells with dosages of vector and miRNA set to 10 mM and 50 mM, respectively. Total RNA isolation was performed every 24 h, followed by RT-qPCRs to confirm the overexpression.

### Preparations of RNA samples

Total RNA isolations and purifications were carried out using the EZ-RNA Total RNA Isolation Kit (Bioind). To effectively isolate high quality RNA, 10 volumes of lysis buffer was mixed with 1 volume of cultivated cells (10^7^) or tissue powder (0.5 g tissue ground in liquid nitrogen). After DNase I digestion of genomic DNA, RNA samples were subjected to Bioanalyzer to evaluate integrity and purity. Bioanalyer results showed that all RNA samples had an RNA integrity number (RIN) higher than 9.0 and a concentration higher than 1,500 ng/ μl.

### Analysis of gene expression with RT-qPCRs

With 3,000 ng of total RNAs as the template, cDNA samples were prepared in a 20 μl reaction system using the SCRIPT cDNA Synthesis Kit (Jena Bioscience, Jena, Germany). Poly-A tail addition was performed. Briefly, total RNAs samples and oligo (dT) primers were mixed, followed by the addition of RNase-free water to prepare a 10 μl mixture, which was then incubated at 65°C for 10 min. After that, 10 μl of the reaction mix was added to prepare a 20 μl mixture, which was then incubated at 50°C for 60 min, and then 70°C for 10 min. With cDNA samples as template, qPCRs were performed to determine the expression of TRERNA1 and miR-23a. Ct values of TRERNA1 and miR-23a were normalized to the internal controls 18S rRNA and U6.

### RNA-RNA interaction analysis with pull-down assay

*In vitro* transcription of both negative control (NC) RNA and TRERNA1 were performed using MEGAscript™ T7 Transcription Kit (Thermo Fisher Scientific) with a T7 vector expression NC RNA or TRERNA1 RNA as template. RNA 3ʹ-end biotin labeling was performed using the 3ʹ Biotin End Labeling kit (Pierce, Rockford, IL, USA). The labeled RNA was named Bio-TRERNA1 or Bio-NC, and was transfected into cells. Cell lysis was performed using lysis buffer on ice 12 h later. After centrifugation, the supernatant of cell lysates was collected, followed by the addition of M-280 streptavidin magnetic beads (Sigma). To avoid nonspecific binding, beads were pre-coated with non-RNase BSA or yeast tRNA prior to use. Beads were collected and washed with low-salt buffer to purify RNA complex. After that, RNAzol was used to isolate RNA samples, which were then used to perform RTs and qPCRs to detect the expression of miR-23a.

### Cellular fractionation assay

Cell fractionation kit (ab109719, Abcam) was used to separate nuclear and cytoplasm fractions of KGN cells. In brief, 1 ml of cell fractionation buffer was mixed with 10^7^ cells, followed by centrifugation at 1,000 g for 10 min. The supernatant, which was cytoplasm fraction, was collected and directly used for RNA isolations. Cell pellet, which was the nuclear fraction, was subjected to further nuclear lysis prior to RNA isolations. Isolated RNA samples were subjected to RTs and PCRs to determine the expression of TRERNA1. PCR products were first separated using 1% agarose gels, followed by staining with EB. The MyECL imager was used to record images.

### Cell apoptosis analysis

At 48 h post-transfection, KGN cells were collected after centrifugation, followed by resuspension in non-serum free medium. Cells were cultured for another 48 h and then harvested after centrifugation. After washing with PBS, cells were stained with fluorescein isothiocyanate (FITC) labeled Annexin-V and propidium iodide (PI). After that, flow cytometry was performed to analyze cell apoptosis.

### Statistical analysis

The GraphPad Prism 6 Software (GraphPad) was used to analyze data. All data were presented as the mean ± standard deviation (SD) from three independent groups. Comparisons among multiple groups were performed using ANOVA Tukey’ test. Comparisons between two groups were performed using Student’s t test. Differences were considered as statistically significant when *p* < 0.05.

## Results

### The expression of TRERNA1 and miR-23a in POF

**Figure 1.**
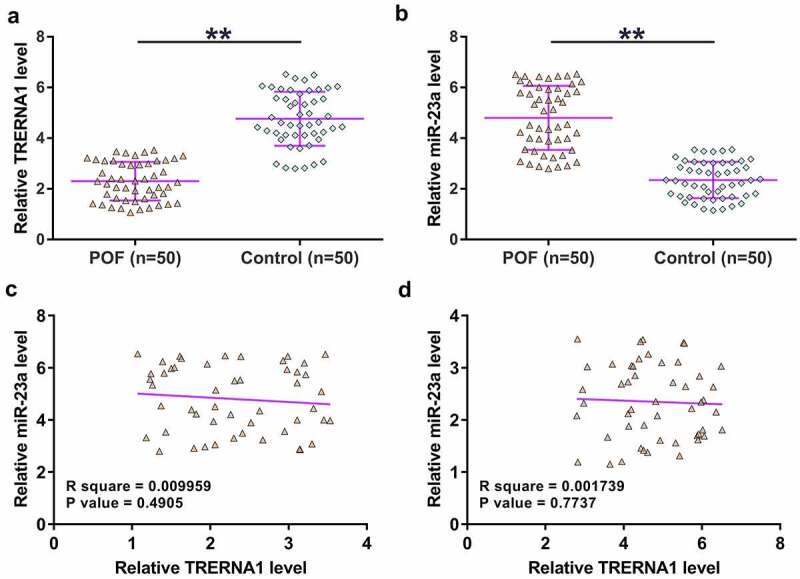
The expression of TRERNA1 and miR-23a in POF. GC tissue samples donated by both POF patients (n = 50) and controls (n = 50) were analyzed through RNA isolation, followed by RTs and qPCRs to determine the expression of TRERNA1 (A) and miR-23a (B). Each dot represents an average value of three qPCR replicates. Correlations between TRERNA1 and miR-23a across POF samples (C) and control samples (D) were analyzed with Pearson’s correlation coefficient. **p < 0.01.

Differential expression of genes was firstly detected. The expression of TRERNA1 and miR-23a in GC tissue samples donated by both POF patients (n = 50) and the controls (n = 50) were determined by qPCRs. The results showed that TRERNA1 was significantly downregulated in POF (Figure 1(a), *p* < 0.01), and miR-23a was upregulated in POF (Figure 1(b), *p* < 0.01). Correlations between TRERNA1 and miR-23a across POF samples and the control samples were analyzed with Pearson’s correlation coefficient. It was observed that TRERNA1 and miR-23a were not closely correlated with each other across POF (Figure 1c) and control (Figure 1(d)) samples. Therefore, TRERNA1 and miR-23a may participate in POF, and TRERNA1 was unlikely a target of miR-23a.

### The subcellular location of TRERNA1 in granulosa cells and its interaction with miR-23a

**Figure 2.**
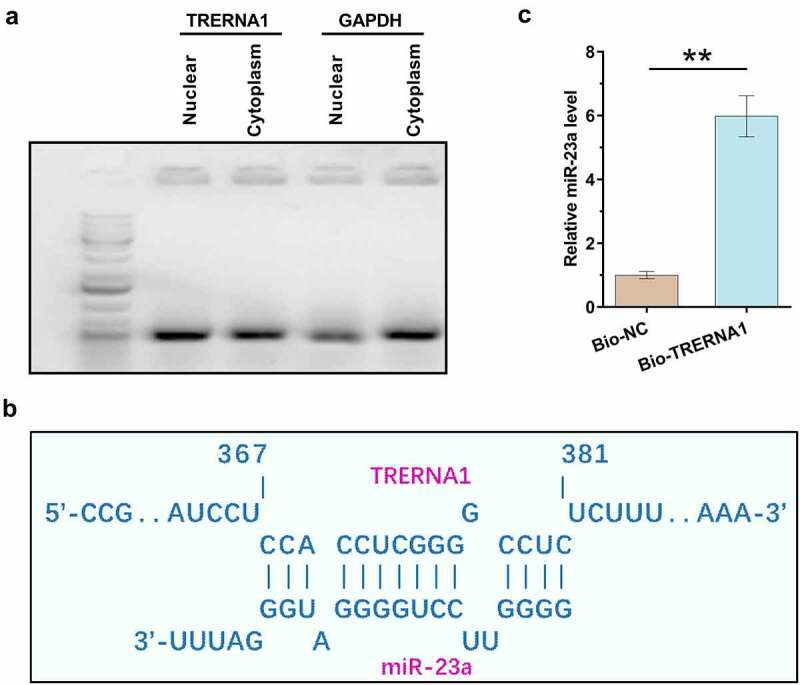
Detection of TRERNA1 in the subcellular location of granulosa cells and analysis of its interaction with miR-23a. Subcellular fractionation assay was performed to analyze the subcellular location of TRERNA1 in both nuclear and cytoplasm fractions of KGN cells. Two fractions were subjected to RNA isolation, followed by RT-PCR to detect TRERNA1. PCR products were subjected to 1% gel electrophoresis. Images were taken after ethidium bromide staining (A). IntaRNA 2.0 was applied to predict the potential interaction of TRERNA1 with miR-23a (B). RNA-RNA pulldown assay was performed to confirm the interaction between them (C). Data presented were values of mean ±SD of three biological replicates. **p < 0.01.

Subcellular location determines function. Subcellular fractionation assay was therefore performed to determine the subcellular location of TRERNA1 in both nuclear and cytoplasm fractions of KGN cells. It was observed that TRERNA1 could be detected in both nuclear and cytoplasm fractions of KGN cells, and the accumulation levels of TRERNA1 in two fractions were similar (Figure 2(a)). In contrast, although GAPDH can be detected in nucleus, it was enriched in cytoplasm. IntaRNA 2.0 was applied to predict the potential interaction of TRERNA1 with miR-23a (Figure 2(b)). It was observed that TRERNA1 and miR-23a could form potential base-pairing. RNA-RNA pulldown assay was performed to confirm the interaction between them. Compared to Bio-NC pulldown sample, Bio-TRERNA1 pulldown sample showed significantly increased expression levels of miR-23a (Figure 2(c), *p* < 0.01). Therefore, miR-23a could directly bind to TRERNA1.

### The role of TRERNA1 and miR-23a in regulating the expression of each other

**Figure 3.**
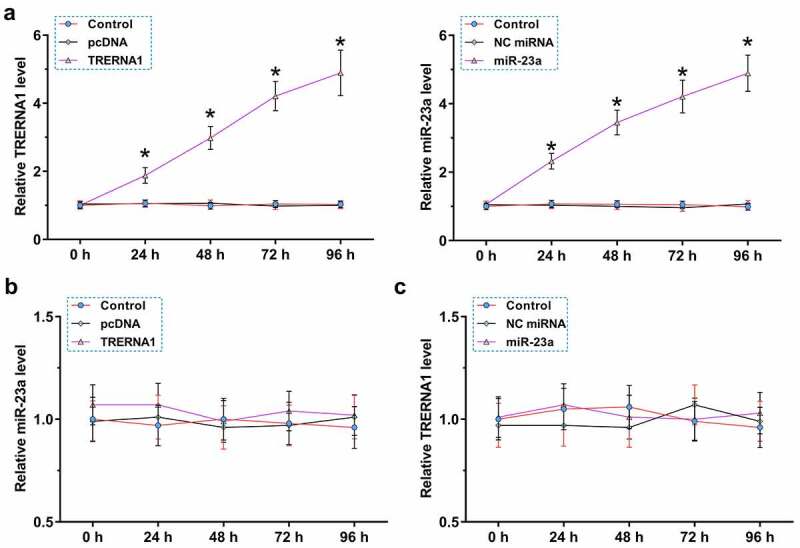
The role of TRERNA1 and miR-23a in regulating the expression of each other. KGN cells were transfected with TRERNA1 expression vector or miR-23a mimic, and their overexpression was confirmed every 24 h until 96 h. It was observed that TRERNA1 and miR-23a were significantly overexpressed during this time period (A). RT-qPCRs were performed to analyze the role of TRERNA1 in the expression of miR-23a (B) and the role of miR-23a in the expression of TRERNA1 (C). Data presented were values of mean ±SD of three biological replicates. *p < 0.05.

To further explore the interaction between TRERNA1 and miR-23a, KGN cells were transfected with TRERNA1 expression vector or miR-23a mimic, and the overexpression was confirmed every 24 h until 96 h. It was observed that TRERNA1 and miR-23a were significantly overexpressed during this time period (Figure 3(a), *p* < 0.05). TRERNA1 did not affect the expression of miR-23a (Figure 3(b)). Similarly, miR-23a also did not affect the expression of TRERNA1 (Figure 3(c)). Therefore, TRERNA1 and miR-23a could not regulate the expression of each other.

### The role of TRERNA1 and miR-23a in KGN cell apoptosis

**Figure 4.**
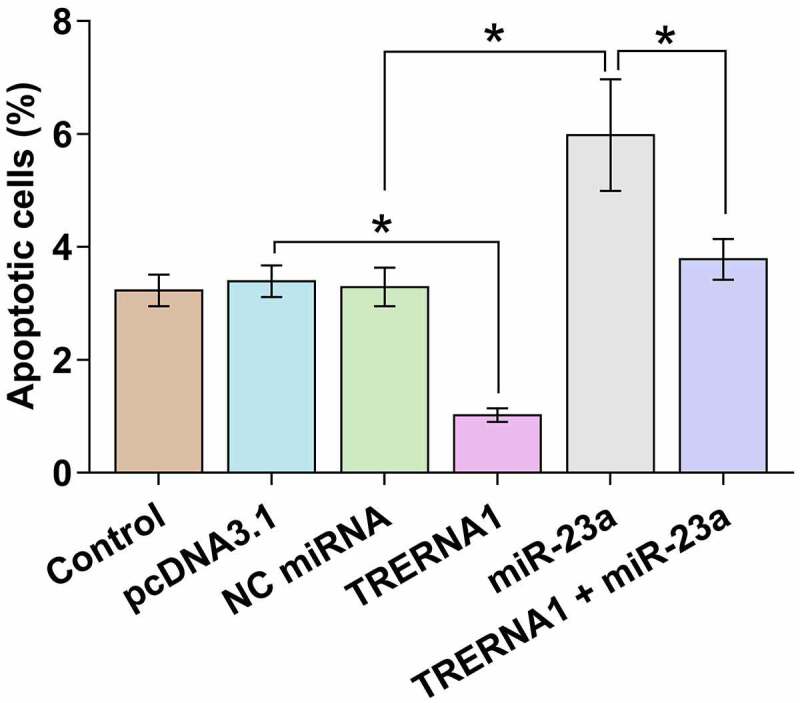
Analysis of the role of TRERNA1 and miR-23a in KGN cell apoptosis. KGN cell apoptosis after the overexpression of TRERNA1 and/or miR-23a was analyzed with cell apoptosis assay.Data presented was values of mean ±SD of three biological replicates. *p < 0.05.

Cell apoptosis contributes to POF. KGN cell apoptosis after the overexpression of TRERNA1 and/or miR-23a was detected by cell apoptosis assay. TRERNA1 decreased cell apoptosis, while miR-23a increased cell apoptosis. TRERNA1 suppressed the role of miR-23a in enhancing cell apoptosis (Figure 4, *p* < 0.05). Therefore, TRERNA1 may regulate cell apoptosis in POF through miR-23a.

## Discussion

The participation of TRERNA1 and miR-23a in POF was explored in this study. We showed that the expression of TRERNA1 and miR-23a were both altered in POF. Moreover, TRERNA1 and miR-23a may interact with each other to regulate the apoptosis of granulosa.

LncRNAs have been demonstrated to play important roles in the pathogenesis of cancers [20]. Previous studies have characterized TRERNA1 as a critical player in cancer biology [16,17]. TRERNA1 is highly expressed in gastric cancer, and it enhances the expression of SNAI1 to increase epithelial-mesenchymal transition, thereby promoting tumor metastasis [16]. TRERNA1 is also upregulated in liver cancer, and reduces the H3K9 methylation of the promoter region of CDH1 genes to regulate the EHMT2/SNAI1 complex, resulting in the increased tumor metastasis [17]. However, the role of TRERNA1 in other types of human disease is unclear. This study showed decreased expression levels of TRERNA1 in POF. Granulosa cells produce LH receptors and steroids to maintain the normal function of ovaries, and increased apoptosis of granulosa cells contributes to the development of POF by reducing ovarian function [21]. In this study we observed that overexpression of TRERNA1 decreased the apoptosis of KGN cells. Therefore, TRERNA1 may participate in POF by suppressing granulosa cell apoptosis, and its overexpression may serve as a potential target to treat POF.

Studies have shown that abnormal expression of miRNAs can be used as potential biomarkers for different types of disease [22]. For example, miR-21 suppressed ovarian granulosa cell proliferation by targeting SNHG7 in premature ovarian failure with polycystic ovary syndrome [22]. In this study, it was observed that miR-23a could promote the apoptosis of granulosa cells, indicating the involvement of miR-23a in POF [18]. This study confirmed the upregulated expression of miR-23a and its enhancing effects on the apoptosis of granulosa cells. However, the upstream regulators of miR-23a in the apoptosis of granulosa cells are unclear. In this study we observed the direct interaction between miR-23a and TRERNA1, which can be detected in both nuclear and cytoplasm samples of KGN cells. Interestingly, although TRERNA1 and miR-23a did not affect the expression of each other, TRERNA1 suppressed the role of miR-23a in promoting KGN cell apoptosis. We therefore speculated that TRERNA1 may sponge miR-23a in KGN cells to suppress cell apoptosis.

## Conclusion

TRERNA1 is downregulated in POF, while miR-23a is upregulated in POF. TRERNA1 may sponge miR-23a to suppress the apoptosis of granulosa cells. This study characterized a novel TRERNA1/miR-23a in POF. TRERNA1 and miR-23a may serve as the targets for the treatment of POF.

## Data Availability

The materials and data used in this research are available after consulting the corresponding author with reasonable requests.
